# A cross-sectional analysis of women’s mental health problems: examining the association with different types of violence among a sample of Brazilian mothers

**DOI:** 10.1186/1472-6874-13-20

**Published:** 2013-04-15

**Authors:** Joviana Avanci, Simone Assis, Raquel Oliveira

**Affiliations:** 1 Latin-American Center of Studies of Violence and Health Jorge Careli (National School of Public Health)/Oswaldo Cruz Foundation, Avenida Brasil 4036 sala 700, Manguinhos, Rio de Janeiro, Brazil; 2 Institute of Clinical Research Evandro Chagas/Oswaldo Cruz Foundation, Avenida Brasil 4036, Manguinhos, Rio de Janeiro, Brazil

**Keywords:** Women, Violence, Mental health

## Abstract

**Background:**

Mental health problems are the major cause of disability in poor countries, and women are the individuals most affected. The World Health Organization points out that violence against women is the leading cause of mental health problems. This study seeks to identify explanatory factors for women’s mental health problems, highlighting situations of violence suffered by them during childhood, when living with a partner and in the community.

**Method:**

A cross-sectional analysis was conducted with 389 mothers with schoolchildren in a city in the state of Rio de Janeiro (Brazil). Profile variables and childhood and adult life experiences were researched and organized in three analytical blocks. A binary logistic regression model was used, divided into hierarchical blocks.

**Results:**

The final model shows that women who were the victims of severe physical violence by their partner were more likely (OR = 8.2) to suffer from mental health problems than those who had never been exposed to this type of violence. The mothers of children with behavior problems are more likely to have mental health problems (OR = 3.0) than mothers whose children do not manifest behavioral problems.

**Conclusion:**

This study shows that women’s mental health problems are particularly related to the experience of physical violence, especially that occurring in intimate partner relationships. Based on this premise, this work recommends that multidimensional issues need to be included in women’s health assistance programs duly incorporating the specificity of victimization by violence.

## Background

Mental health problems (MHP) are the most important cause of disability in poor countries, accounting for 12% of the total disease burden, with an estimate of 15% by 2020 [1]. Despite its importance, it is the most overlooked problem in global health policy [2]. Anxiety and depression are the most prevalent mental health problems and women are the individuals most affected (about three times more than men) [3].

In addition to biological factors, there is the difficult social condition of women, who mostly tend to perform informal jobs and have lower social status and income than men for the same job, which is one of the aspects that make women more likely to develop mental health problems [4]. Moreover, other gender issues are involved, notable among which are the lack of social incentives for autonomy in decision-making, lack of support for child care, daily chores and limited access to healthcare services, education or a social support network [2,5]. Chronic poverty conditions, drug use by family members and some temperamental characteristics are other factors that exacerbate women’s mental health problems [4].

Combined with this, there is the problem of motherhood, as especially in developing countries where the responsibility to care for and meet children’s needs (emotional, educational, financial and leisure) falls on women, many of whom live alone with their children. In worldwide terms, approximately 70% of the 1.2 billion people living in poverty are women living alone or with dependent children [4]. In general, women work double or triple shifts, with a significant accumulation of responsibilities and functions. For the mother, life can become even more complicated when the child has severe physical or cognitive limitations or even behavioral problems.

Among the factors associated with women’s mental health problems, violence experienced in childhood and adulthood has received special attention from specialists [6,7]. The World Health Organization [1] points out that violence among women is the leading cause of mental health problems leading to an increase in the consumption of tranquilizers and antidepressants in this group [8].

There are high rates of violence worldwide inflicted upon women since childhood. It is estimated that 55% of women have experienced some type of violence in their lives [9]. In childhood, violence estimates are around 7% to 40% [10,11] and, in adulthood, 10% to 56% of women reported physical abuse from their partner at least once in their lifetime [12,13]. Moreover, the probability of re-victimization by violence in adulthood is high, such that women with a history of physical or sexual violence in childhood tend to be two or three times more exposed to intimate partner violence than those who did not suffer abuse as children [14,15]. Women are also affected by violence in the communities in which they live, such as drug trafficking, assaults and robberies, kidnappings, murders and armed conflicts, which significantly affect their daily lives, especially those living in poorer areas. Fear and insecurity generated in these conditions can cause mental problems, exacerbating vulnerabilities both in the personal and in the family context.

However, there are still many lacunae to be addressed regarding violence and women’s mental health, especially due to the tendency of studies to prioritize most isolated aspects, without, however, discussing them in a more contextual manner. The majority of studies investigate the following lines of research: the immediate and subsequent consequences of violence, especially sexual abuse; the effects of intimate partner violence; and the period when violence took place in the woman’s life, as childhood, pregnancy and the postpartum phases are particularly sensitive. Specifically, the lacunae include the cumulative effect of abuse; the indirect effect of violence, including witnessing violence; and violence experienced away from home.

Based on a comprehensive approach, the World Health Organization proposes an understanding of violence based on the ecological model, which is the result of systemic action of many factors that are organized at the individual, relationship, community and social levels [16]. This approach is consistent with the Bioecological Theory of Human Development [17] and the analytical proposal of Victora et al. [18], which examines the individual in a system, including proximal aspects (which affect the outcome studied more directly), undergoing the intermediate variables (acting through a number of inter-related variables) to the distal variables (which rarely cause ill-health directly). Based on that, the purpose of this paper is to identify explanatory factors of women’s mental health problems, mostly the situations of violence they experienced in childhood, living with a partner and living in their community, which may encourage the debate on prevention and on public policies aimed at women.

## Methods

### Participants

This study is based on longitudinal research started in 2005, originally with 500 schoolchildren attending the second year of basic education of a public system school in a city located in the state of Rio de Janeiro/Brazil (São Gonçalo). The city shows great social poverty, with low per capita income (US$154.32 in 2000) [19]. In the city,violence and accidents rank in fourth place among the causes of morbidity and mortality of women ranging from 20–59 years old: 7,3% of all deaths in 2010 [20]. The sample design used is of the conglomerate type in three selection stages: schools, classes of year attended and students. Students in the classes were selected by simple random sampling.

This article is based on cross-sectional analysis of the third wave of research (2008) and includes 389 women responsible for the schoolchildren studied (53 dropped out of the study and the remainder include the participation of informants other than the mother).

The data analyzed in this paper include the population of students from the public school in the city studied. Thus, weights for each student selected for the study were calculated, according to the allocation in each of the sampling units (school, class, and students). Therefore, all data from mothers were weighted according to the sample weight calculated for the students, considering all stages of selection. The total of mothers of children used in the analysis included 5069 participants.

### Ethical considerations

All researchers underwent training to improve the quality of their data collection skills by including knowledge about violence, and skills on how to communicate empathetically and how to maintain privacy and security during interviews. Interviewers informed each respondent of their right to refuse to participate, and of their right to refuse to answer any question. Before starting with the questionnaire, the interviewers requested verbal consent to proceed. The study was approved by the Ethics Committee in Research of the National School of Public Health/Fiocruz, and the women signed terms of free and informed consent.

### Measures

The mothers of the children selected were interviewed individually by the trained researchers, filling out a multidimensional questionnaire. The following explanatory variables were investigated and divided into three analytical blocks, according to a hypothetical strength relation (distal to proximal impact) [17,18].

*Socio-economic profile (Distal block).* This block includes the following questions: a) social strata of the family, which was estimated according to the family’s assets and the head-of-family’s schooling, scored as B/C1/C2 (*upper/middle social strata*) and D/E (*lower social strata*) [21]; b) family income, estimated from the total amount of wages and other payments received by the family, and social benefits/pensions. It was classified by the minimum wage (mw) in 2008, which was equivalent to $250, and categorized in five variables: income < 1/4 mw (< US$62.5), 1/4-1/2 mw (US$62.5-US$125), 1/2-1 mw (US$125-US$250), 1 to 2 mw (US$250-US$500) and 2 to 5 mw (US$500-US$1250); c) family structure, characterized by the people the woman lives with; d) women’s education, classified into two groups (literate and illiterate); e) number of children living in the same house; and f) type of work performed by the woman, with the type of employment bond being prioritized (Table 1).

**Table 1:** Distribution of variables of the socio-economic profile block, according to women’s mental health problems, São Gonçalo/RJ/Brazil (Distal block)

**Variables**	**Categories**	**No MHP**	**With MHP**	p-value*
Social Strata (N-4001)	B (upper social strata)	4.9	2.6	0.272
	C1 (middle social strata)	37.3	29.6	
	C2 (middle social strata)	37.8	41.8	
	D/E (lower social strata)	20.0	26.0	
Family Income (minimum wage ranges) (N = 5002)	< 1/4 mw (US$62.5)	22.2	26.8	0.22
	1/4-1/2 mw (US$62.5- US$125)	39.1	43.5	
	1/2-1 mw (US$125 -US$250)	30.5	23.5	
	1 to 2 mw (US$250-US$500)	5.2	5.5	
	2 to 5 mw (US$500-US$1250)	3.1	0.7	
Woman’s Family Structure (N = 4788)	Lives by herself	5.1	4.2	0.879
	Lives with children (without partner)	28.1	30.9	
	Lives with children and new partner	16.6	17.6	
	Lives with children and partner (children’s father)	50.2	47.2	
Women’s education (N = 5069)	Illiterate	3.0	7.4	0.08
	Literate	97.0	92.0	
Number of children living in the same house (N = 5069)	≥ 2 children	53.1	50.1	0.528
	< 2 children	46.9	49.9	
Type of work (N = 5042)	Employee/Employer/Retired	35.7	27.0	**0.025**
	Self-employed	30.4	28.4	
	Unemployed/Does not work	33.9	44.6	

* p-value <0.05.

*Experiences in childhood (Intermediate block).* All issues investigated in this block refer to the women’s childhood and were surveyed in 2006: a) women witnessing violence between their parents in childhood, to the point of getting hurt and/or humiliating each other; b) situation of beating of the woman (interviewed mother) by her parents when she did something wrong; c) if she was severely beaten by her parents; and d) woman’s self-image as a child, if she was happy, sad/depressed, agitated/hyperactive, disobedient, aggressive, fearful and/or anxious (Table 2).

**Table 2:** Distribution of variables of the childhood experience block, according to women’s mental health problems, São Gonçalo/RJ/Brazil (Intermediate block)

**Variables**	**Categories**	**No MHP**	**With MHP**	p-value*
Witnessed violence between parents (N = 4149)	Yes	26.8	35.9	0.167
	No	73.2	64.1	
Parents had beaten the woman when she did something wrong (N = 4496)	Yes	62.6	76.8	**0.015**
	No	37.4	23.2	
Parents had beaten the woman severely (N = 4522)	Yes	10.9	21.9	**0.023**
	No	89.1	78.1	
Self-image as happy (N = 4548)	Yes	83.1	66.4	**0.004**
	No	16.9	33.6	
Self-image as sad/depressed (N = 4562)	Yes	28.4	47.8	**0.001**
	No	71.6	52.2	
Self-image as agitated/hyperactive (N = 4575)	Yes	37.8	54.5	**0.001**
	No	62.2	45.5	
Self-image as disobedient (N = 4508)	Yes	33.4	39.7	0.314
	No	66.6	60.3	
Self-image as aggressive (N = 4548)	Yes	5.3	16.6	**0.001**
	No	94.7	83.4	
Self-image as fearful (N = 4548)	Yes	56.2	58.7	0.63
	No	43.8	41.3	
Self-image as anxious (N = 4481)	Yes	58.7	73.0	**0.044**
	No	41.3	27.0	

*p-value <0.05.

*Adulthood experiences (Proximal block).* The following items were evaluated: a) total behavioral problems of her child, investigated using the CBCL (Child Behavior Checklist) [22,23], which is characterized by internalizing problems (anxiety/depression, withdrawal/depression and somatic complaints), externalizing problems (rule-breaking and aggressive behavior), attention problems, thinking problems and social competence of the child. The T score index was applied for defining the groups: non-clinical (T < 65), borderline (T = 65-69)/clinical (T > 69). Cronbach’s α of 0.92; b) support from close friends/family with whom the woman feels comfortable to talk; c) external support for women (church, community, health services, etc.); d) participation in neighborhood associations; e) involvement of family members with alcohol or drugs; f) victim of severe physical violence perpetrated by the intimate partner (Conflict Tactics Scale, CTS-1) [24], with the following actions being investigated: kicking, biting or punching, harshly beating, threatening or using a weapon such as gun or knife. Cronbach’s α of 0,82; g) rates of community violence rates registered through occurrence records between 07/2007-06/2008, based on a survey held in the database of the Civil Police of the State of Rio de Janeiro/State Department. The report of an incident is the document that starts the process and registers, in the Civil Police Department, the first information about the crime, which serves to open an investigation and gather evidence for instructing the process that will lead to the sentence. Based on these data and population estimates for 2008, the rates for threat, theft, homicide, bodily harm and possession or illegal use of drugs and weapons were calculated. The rates were calculated per 1000 inhabitants. The population rates were analyzed according to the neighborhood of the woman participating in the research and according to the groups studied (with or without mental health problems) and are presented as mean and standard deviation (Table 3).

**Table 3:** Distribution of variables of adulthood experiences block, according to women’s mental health problems, São Gonçalo/RJ/Brazil (Proximal block)

**Variables**	**Categories**	**No MHP**	**With MHP**	p-value*
Total children’s behavioral problems (N = 5069)	Non-clinical	94.0	74.3	**0.001**
	Borderline/Clinical	6.0	25.7	
Support from close friends/family (N = 5029)	Yes	73.4	69.6	0.354
	No	26.6	30.4	
External Support (N = 5042)	Yes	41.7	43.9	0.722
	No	58.3	56.1	
Participates in neighborhood associations (N = 5029)	Yes	8.7	9.5	0.765
	No	91.3	90.5	
Severe physical violence of intimate partner (N = 3309)	Yes	2.0	11.1	**0.008**
	No	98.0	88.9	
Family member with an alcohol or drug problem (N = 5029)	Yes	18.1	33.8	**0.005**
	No	81.9	66.2	
Threat rate (N = 4656)	Mean+/− standard deviation	3.97+/−0.28	3.95+/−0.30	0.945
Theft rate (N = 4656)	Mean+/− standard deviation	7.77+/−0.81	7.38+/−0.70	0.598
Homicide rate (N = 4656)	Mean+/− standard deviation	0.93+/−0.23	0.98+/−0.17	0.799
Bodily harm rate (N = 4656)	Mean+/− standard deviation	5.02+/−0.36	4.98+/−0.42	0.941
Possession or illegal use of drugs and weapon rate (N = 4656)	Mean+/− standard deviation	0.68+/−0.10	0.64+/−0.08	0.680

*p-value <0.05.

To evaluate the *women’s mental health problems*, the Self-Reported Questionnaire - SRQ20 was applied [25], which has 20 dichotomous items, including symptoms of anxiety, depression, and somatic complaints. The SRQ-20 has shown to be comparable with the General Health Questionnaire (GHQ-12) [26] and has proven very effective in screening for non-psychotic disorders. To analyze the scale, all items were totted up. The women who scored 8 or more points were considered to suffer from a mental health problem. Cronbach’s alpha is 0,854.

Almost all scales used in this study obtained adequate and satisfactory psychometric results in this study and in others carried out in the country and abroad [19,27,28].

### Data analysis

The descriptive data analysis examined the frequencies and description of the variables investigated. The association between categorical variables and mental health problems was verified by the Rao-Scott chi-square independence test, with second order correction. The *t*-test was used to identify the differences in mean values of rates according to the problem investigated (alpha level of .05).

All the variables considered statistically associated with mental health problems were entered into a binary logistic regression model in accordance with the three hierarchical blocks (Socio-economic Profile, Childhood Experiences, and Adulthood Experiences) [18,27,29,30]. Thus, three successive models were prepared for each block, always considering the significant variables in the previous model. The Wald test, at 5% level, was used to select the variables that would remain in each block of the model studied, since the input of the variables in the levels of analysis (blocks) was performed manually. The quality of the model is informed by the Akaike (AIC) criteria (the lower value indicates the best adjustment).

The analysis described was performed in the Complex Samples module of the Statistical Package for Social Sciences – SPSS version 16.0 software (Chicago, USA). All the analyses included the expansion of data by sample weight, and correction of the variance by the sampling design used.

## Results

Of the mothers, 38.9% manifested mental health problems. As for the socio-demographic profile, almost all of the women belonged to the lower classes (C/D/E) (96%); most of them (92.4%) have a family income up to US$ 250; 65.1% live with the child’s father or another partner; 52.2% have more than 2 children and 4.6% are illiterate. Sixty-two percent of women were partially employed, formally or informally, while the remainder did not work.

In their childhood, women picture themselves mostly as happy (76.9%), anxious (64.4%), fearful (57.7%), restless/hyperactive (44.7%), disobedient (35.8%), sad/depressed (35.7%) and aggressive (9.5%). Thirty percent (30.5%) of the women reported witnessing violence between their parents, 68.5% said that their parents would spank them as children and 14.8% reported victimization with severe beating by their parents.

In adulthood, when evaluating the behavioral problems of their children, 14.1% of them reported some type of problems, at borderline or clinical level. Most women reported the existence of social support (71.5%). With respect to episodes of violence in adulthood, 6% reported experiencing severe physical violence from their intimate partner and 24.1% had a family member involved with drugs. In the overall analysis on community violence where the women live, the average theft rate was of 7.60/1,000 inhabitants, the bodily harm rate was 4.99/1,000 inhabitants, the threat rate was 3.95/1,000 inhabitants, the homicide rate was 0.94/1,000 inhabitants, and the average of illegal possession or use of drugs and weapons was 0.67/1,000 inhabitants.

Tables 1, 2 and 3 show the occurrence of mental health problems among women according to the socio-economic profile variables, childhood experiences and, finally, more proximal variables, experienced in adulthood. Out of the 27 variables studied, only 11 variables are associated with women’s mental health problems.

In the first block (Table 1), only the relationship on the type of work performed by the woman showed a statistical association with mental health problems, as there were more women with problems investigated among the unemployed and those who do not work (44.6%). Social strata, family income, family structure, women’s education, and number of children at home were not associated with the outcome studied.

The second block (Table 2) reveals a higher prevalence of severe physical violence experienced in childhood and signs of emotional difficulties as children in the group of women with mental health problems. About 22% of them with mental health problems report that their parents had beaten them severely as children, while 11% of the group of women without problems investigated reported experiencing such violence. Similarly, 76,8% of women with mental health problems reveal that parents had beaten them in childhood when she did something wrong, otherwise 62,6% without problems studied reported it. Signs of sadness, agitation, aggression, and anxiety in childhood are more frequently reported by women with mental health problems, compared to the other group. Almost 34% of women who manifested mental health problems say they were not happy as children, which occurs in about 17% of those from the other group.

In the final block (Table 3), severe violence committed by the intimate partner, family member involvement with alcohol and drugs, and mental health problems of the child are variables associated with women’s mental health problems. All these issues were mostly reported by those women with problems investigated. In the group of women with mental health problems, 11.1% of them claimed to have suffered severe physical violence from their intimate partner (versus 2% in the control group), 33.8% reported family member involvement with alcohol or drugs (versus 18.1%) and 25.7% of the children also had behavioral problems (versus 6% of the other group).

In the final hierarchical logistic regression tested with the 11 selected statistically significant variables in the exploratory analysis, only two remained in the final model: severe physical violence with intimate partner and child behavioral problems (Table 4). No interaction among the variables was significant.

**Table 4:** Final model of hierarchical logistic regression for women’s mental health problems, São Gonçalo/RJ/Brazil (N = 2682)

**Variables**	**Categories**	**Crude OR**	**Adjusted OR**	**CI (OR) 95%**
Severe physical violence of intimate partner	Yes	6.1	8.2	1.5-45.15
	No	1.0	1.0	-
Total behavioral problems of children	Borderline/Clinical	5.4	3.0	1.33-6.63
	Normal	1.0	1.0	-

Women who were victims of severe physical violence from their intimate partners were more likely (OR = 8.2, CI 1.5-45.15) to have mental health problems than those who had never suffered this type of violence. The mothers of children with behavioral problems are more likely to have mental health problems (OR = 3.0, CI 1.33-6.63) than those without children with behavioral problems.

## Discussion

The results add to and reinforce the finding of a strong relationship between violence experienced by women and mental health problems. Thus, the study contributes to the discussion of women’s mental health related to several forms of violence experienced during childhood and adulthood, which occurred within the family and the community.

In the bivariate analysis, the lack of an association between women’s mental health and family income, social status and educational level of women stand out. However, unemployment proves to be an important aspect, which can be hypothesized by three aspects, which also explains the lack of association with the variables mentioned above. First, the lack of money, regardless of amount, is more important than the general socio-demographic profile. Secondly, it is common knowledge that the sense of non-productivity, dissatisfaction, and financial dependence of housewives can impact their mental health. Furthermore, staying at home, their universe of emotional support and information is reduced, leading to increased susceptibility to mental problems. Third, the group investigated shows a very similar socio-economic profile, not enabling comparisons between disparate socio-economic realities. Thus, unemployment might be a variable that enables a more precise assessment of the social status of these women that is related to their mental health. For Patel and Kleinmain [31], poverty itself can only partially explain the greater vulnerability of the poor to psychiatric disorders, and other individual and social characteristics that are greater determinants [32]. Regarding the lack of association between women’s mental health with family structure and social support, the methodological aspects (not using the standardized scale to assess social support) and socio-cultural aspects (great mobility in the family setting) can support the discussion.

Regarding the core purpose of the study, the relationship between violence and women’s mental health problems is emphasized. Despite only being observed in bivariate analysis that violence experienced in childhood is linked to mental health problems, it is important to draw attention to this link due to the harm caused by such violence in later years, affecting the way that women feel and relate to others. As observed by Koss et al. (2003) there is also the lack of support from health and social services and the invisibility of violence and its consequences to health [33]. The present study further highlights the association between mental health problems and victimization by violence perpetrated by the intimate partner, which is consistent with previous studies [6,34-36]. Romito et al. [36] state that the social determinants of women’s health do not in general include violence in their health policy protocols, which affect the approach to the problem. In Brazil and other countries, although significant progress has been made, even in the legislative sphere, many health professionals are still reluctant to provide assistance in cases of violence, which contributes to the perpetuation of the problem and the severity of its effects [35].

From a more systemic approach, which renders the violence issue even more serious, there is a tendency for the continuity and simultaneity of victimization with different types of violence throughout life, as each one of them can overlap, impacting health differently as well as having a cumulative effect. At this point, the continuity of violence experienced by the women surveyed in childhood and adulthood, resulting in an accumulation of risk over time, should be examined. Moreover, although violence in the community has not been revealed to be related to women’s mental health problems, structurally disorganized communities elicit offending and delinquent behavior, which encroaches upon the private space and closer relationships [37]. Raghavan et al. [38] use the following arguments to explain the close relationship between violence between the couple and the community: (1) the lack of collective efficacy of women with mental health problems who, when faced with reduced social involvement, tend to have greater tolerance to violent and undesirable behavior between the couple; (2) the fear of crime, such that when women protect themselves from community violence, they isolate themselves from neighbors, restricting their support network; and (3) the acceptance and legitimacy of violence between the couple in communities where violence is considered normal and seen as a way of solving conflicts and of communication. Moreover, the social support of the community may not be able to help women in violent situations with their partners, minimizing the seriousness of the situation, blaming them for what happened and encouraging the couple to remain in the relationship [39-41], contributing to isolation and female submission. Another issue that arises in the context of women’s mental health and violence is the use of drugs, which can increase women’s risk of exposure to violence in the community and with their partners.

Furthermore, the association of women’s mental health problems with their children’s behavior is a recurrent issue observed in other studies [42]. This topic initially draws attention to the high prevalence of mental health problems in the women’s groups studied (38.9%), which is consistent with other Brazilian studies [35], but slightly higher than in other countries, especially in Asian where it is around 20% [6,43]. The sorry condition of women’s mental health includes the difficulty of mothering and the tendency to punish their children as a way of educating or being permissive, leaving the decision on the best behavior up to their children. The sentiment of inadequacy and insecurity felt by mothers with mental health problems is related to a wide range of negative consequences for their children, which may contribute to their problems [44]. The situation is more serious for the child when these problems are not treated and tend to be recurrent, affecting their health though to adulthood. The relationship between the mental health problems of the mother and the child is so close that even biological issues can support the intergenerational discussion.

Finally, reflecting on our findings based on the ecological model proposed by the WHO for understanding violence, two seemingly antagonistic issues can be discussed: (1) that the variables considered to be proximal in this study (severe physical violence of intimate partner and children’s behavior) are superimposed on those studied in the intermediate and distal levels, which indicates the power of the immediate relationship environment on health; (2) that despite the proximal variables having been considered more explanatory for the existence of mental health problems in women, it is important not to overlook the systemic, reciprocal and progressive action, of the biopsychcologically active and evolving human being with people, objects and symbols found not only in their immediate environment [17]. These factors affect dynamically and indirectly how interpersonal relationships occur and may, for example, facilitate violence between the couple [17,45]. Furthermore, application of the hierarchical model is an approximation to the ecological model, which has technical limitations, especially due to the theoretical complexity advocated by the first model. Thus, future research should invest in efforts to use comprehensive methodological approaches that can be aggregated for a better understanding of the phenomenon.

With respect to the limitations of this study, there are many lacunae that need to be studied and elucidated. First, some assessments are retrospective, which can introduce a recall bias. Second, the cross-sectional design does not make it possible to investigate the possibility of reverse causality. Furthermore, the statistics of community violence do not take into account the perception and interpretation of women’s experiences. Another limitation lies in the variety of statistics, which can generate confusion and error, though this was partially minimized by block and univariate analysis. Finally, with respect to the nonspecificity of mental health problems studied, the identification of potential factors may indicate aspects that must be considered in the prevention and treatment of any mental disorders in women.

## Conclusion

This study shows that the mental health problems of women are particularly related to the experience of physical violence, especially that occurring in an intimate partner relationship. Efforts should be made to extend the investigation into the impact of violence on women’s mental health through a qualitative approach. The option to work with the ecological and hierarchical model in this paper adds systemic knowledge of the impact of violence on health, considering more individual aspects as well as relationship and community aspects. Based on that, this work recommends that multidimensional issues need to be involved in the assistance programs of women’s health including the specificity of victimization by violence.

## Competing interests

The authors declare that they have no competing interest.

## Authors’ contributions

JA participated in data collection, conducted the literature research and data analysis, and drafted the article. SA made a substantial contribution to the methodology and interpretation of results and helped draft the manuscript. RO was responsible for the data analysis. All of the authors read and approved the final manuscript.

## Pre-publication history

The pre-publication history for this paper can be accessed here:

http://www.biomedcentral.com/1472-6874/13/20/prepub
